# Effects of BIS076 in a model of osteoarthritis induced by anterior cruciate ligament transection in ovariectomised rats

**DOI:** 10.1186/s12891-015-0547-9

**Published:** 2015-04-17

**Authors:** María Luisa Ferrándiz, María Carmen Terencio, María Carmen Carceller, Ramón Ruhí, Pere Dalmau, Josep Vergés, Eulàlia Montell, Anna Torrent, María José Alcaraz

**Affiliations:** Department of Pharmacology and IDM, University of Valencia, Av. Vicent Andrés Estellés s/n, 46100 Burjasot Valencia, Spain; Technological Extraction Department, BIOIBERICA S.A., Pol. Ind. “Mas Puigvert”, Crta. N-II, Km 680.6, 08389 Palafolls Barcelona, Spain; Pre-Clinical R&D Department, Pharmascience Div., BIOIBERICA S.A., Francesc Macià 7, 08029 Barcelona, Spain

**Keywords:** Osteoarthritis, BIS076, Anterior cruciate ligament transection model, Ovariectomised rats

## Abstract

**Background:**

Osteoarthritis (OA) is the most frequent articular disease and a leading cause of disability. There is a need for effective treatments able to slow the progression of disease. Some of the available treatments are dietary supplements providing natural components. Recent studies have shown that estrogen deficiency contributes to the pathophysiological events of OA progression.

**Methods:**

We have used the anterior cruciate ligament transection model of OA in ovariectomised rats to study the effects of BIS076, a new formulation of a natural porcine cartilage extract associated with hydroxyapatite (as a source of calcium) and vitamin D_3_. Cartilage degradation, proteoglycan depletion and synovitis were followed by histochemistry. Effects on bone microstructure were determined by μCT. The levels of biomarkers in serum and inflammatory mediators in knee homogenates were measured by luminex or ELISA.

**Results:**

Oral administration of BIS076 reduced articular cartilage damage and serum levels of cartilage degradation markers C-telopeptide of type II collagen and cartilage oligomeric matrix protein, as well as matrix metalloproteinase-3. The local inflammatory response was down-regulated by BIS076 with lower production of pro-inflammatory cytokines and prostaglandin E_2_ in joint tissues. In addition, BIS076 was effective on metaphyseal bone alterations as this formulation increased volumetric bone mineral density and improved bone micro-architecture. These effects were related to the modification of bone metabolism reflected by changes in bone biomarkers with reductions in the ratio receptor activator of nuclear factor κB ligand/osteoprotegerin and the levels of tartrate-resistant acid phosphatase-5b, suggesting an inhibitory activity of BIS076 on trabecular bone resorption.

**Conclusions:**

We have demonstrated the protective properties of a new formulation (BIS076) on joint lesion and bone alterations in an experimental model of OA in ovariectomised rats. This study supports the interest of BIS076 in OA treatments.

## Background

Both osteoarthritis (OA) and osteoporosis are major public health problems affecting hundreds of millions of people. Disability related to these conditions is expected to rise as a result of population aging [1,2]. OA, the most common form of rheumatic disease, is characterized by degeneration of articular cartilage, synovitis and changes to subchondral bone which exhibits altered remodelling [3,4]. The development of OA may be related to a combination of local joint-specific factors acting in the context of systemic susceptibility. Numerous systemic factors can influence OA development including genetics, age, gender, sex hormones, dietary intake and bone mineral density, as well as local mechanical factors such as muscle weakness, obesity, and joint laxity [2,5].

Osteoporosis is characterised by low bone mass and micro-architectural deterioration of bone tissue, leading to bone fragility and susceptibility to fracture [6]. Approximately 30% of all postmenopausal women have osteoporosis in the United States and Europe [7]. Age and weight are most closely associated with the development of osteoporosis although a number of other factors are involved such as height, duration of menopause and presence of chronic diseases such as hypertension, hyperlipidaemia, diabetes mellitus, and OA [8].

An inverse relationship between OA and osteoporosis has been debated over years. However, recent studies have revealed several factors which contribute to the pathogenesis of both disorders such as subchondral bone loss or inflammation [9]. Although some studies have reported a relationship between OA and increased bone mineral density [10] this feature is not associated with joint space narrowing [11]. Despite high systemic bone mineral density, bone quality and strength may be decreased at the systemic level in OA of the knee [12]. In addition, a significant decrease in periarticular subchondral bone mineral density is present in female patients with relatively mild OA of the knee [13] and structural OA changes are faster in the presence of local and systemic bone loss [14]. Given the relationship between bone resorption and cartilage damage, inhibition of bone resorption may prevent the progression of OA [15]. In fact, antiresorptive drugs have shown positive effects on bone changes and cartilage destruction in OA [16,17].

Estrogen deficiency is the cause of both the early, accelerated and the late, slow phases of bone loss in postmenopausal women and it also contributes to the continuous phase of bone loss in aging men [18]. Interestingly, epidemiologic OA studies have shown that females after the menopause are affected at a higher rate than males [5,14] and recent evidence indicates that estrogen deficiency plays a key role in the molecular pathophysiological events of joint damage and OA progression [19].

The search for treatments able to slow the progression of disease is a great challenge and would meet an important medical need for patients with OA. The rising incidence of refractory responses to available drugs has fuelled the interest in dietary supplements to provide the natural components that may help to preserve the structural integrity of joint tissues [20]. Anterior cruciate ligament transection (ACLT) in rats is a procedure that induces joint instability leading to a pathological state similar to OA. In addition, recent animal studies have shown that loss of estrogen increases damage in articular cartilage and subchondral bone [21]. In this study, we used the ACLT model of OA in ovariectomised rats to study the effects of a new formulation (BIS076) of a natural porcine cartilage extract associated with hydroxyapatite (as a source of calcium) and vitamin D_3_ on articular degradation and bone changes.

## Methods

### Animals

Female Wistar rats (9 weeks old) were obtained from Janvier (Le Genest Saint Isle, France). The animals were maintained at the animal house of the School of Pharmacy, University of Valencia, Spain, with controlled temperature (21 ± 2°C) and on a 12 h dark–light cycle. Water and food were provided *ad libitum*. All studies were performed in accordance with European Union regulations for the handling and use of laboratory animals. The protocols were approved by the institutional Animal Care and Use Committee (University of Valencia, Spain).

### Ovariectomy and ACLT

Female Wistar rats (180–200 g body weight, 10 weeks old, n = 15 rats/group) were used in this study. Ovariectomy was performed (week 0) and 2 weeks after (week 2) ACLT was performed via an incision on the medial aspect of the right knee joint capsule. All surgical procedures were carried out under deep anaesthesia with isoflurane (1.5%) which was followed by the subcutaneous injection of butorphanol (2 mg/kg). A group of animals (Sham) underwent sham ovariectomy at week 0 followed by sham ACLT at week 2. Another group of rats (Ovx) underwent ovariectomy at week 0 followed by sham ACLT at week 2. Animals were weighed every two days throughout the experiment. There were no significant differences in body weight between groups during the study (data not shown).

### Drug treatment

Rats subjected to ovariectomy + ACLT were divided into three groups: Control and two treatment groups (T1 and T2). BIS076, a preparation of a natural extract from porcine cartilage (containing 55.5% type II collagen and 21.3% glycosaminoglycans, mainly chondroitin sulfate) + hydroxyapatite + vitamin D_3_, was provided by Bioibérica S.A. (Barcelona, Spain). The composition of BIS076 is indicated in Table 1. BIS076 was administered daily (oral gavage of a dispersion in water) at two doses (T1: 163.5 mg/kg and T2: 245 mg/kg) from week 0 until week 12 after ovariectomy. T1 and T2 correspond approximately to daily doses in humans of 1,402 and 2,102 mg, respectively. The amount of vitamin D_3_ in T1 and T2 corresponds to 200 IU in humans. Sham, Ovx and ovariectomy + ACLT (Control) groups, received the same volume of vehicle (water). After week 12, animals were sacrificed. Previously, they were anesthetized with isoflurane and blood was collected by cardiac puncture. All determinations were performed at this time point.

**Table 1:** **Components of BIS076**

	**T1 (low dose)**	**T2 (high dose)**
Porcine cartilage extract	600 mg	900 mg
Hydroxyapatite	800 mg	1,200 mg
Vitamin D_3_	2 mg	2 mg

### Histopathology

Knee joints were removed and fixed in 4% paraformaldehyde in phosphate-buffered saline solution. Samples were decalcified in 10% EDTA, dehydrated in graded ethanol, cleared in toluene and embedded in paraffin. Serial sections were cut (7 μm) and mounted on SuperFrost glass slides (Menzel-Gläser, Braunschweig, Germany). Sections were deparaffinised in xylol, rehydrated in graded ethanol and stained with haematoxylin and eosin (general morphology) or safranin O (proteoglycan). The sections were mounted on a DPX medium (Panreac, Barcelona, Spain) and examined under a light microscope (Nikon Eclipse E800, Izasa S.A., Valencia, Spain) with 10x objective lens and a Nikon Digital Camera DXM1200 using Nikon ACT-1 software. Histopathological grading of cartilage degradation was performed on haematoxylin-eosin and safranin O stained sections by two independent, blinded observers in accordance with the Osteoarthritis Research Society International (OARSI) histopathology grading and staging system [22]. Synovitis was scored according to Krenn *et al*. [23].

### X-ray micro-computed tomography (μCT)

The hind paws were kept wrapped in gauze soaked in 0.9% NaCl at −20°C until analysis by μCT. Samples were analysed with a SkyScan 1172 μCT unit (Bruker microCT NV, Kontich, Belgium). Three-dimensional trabecular micro-architecture was analysed at the metaphysis and epiphysis of the tibia. Samples were imaged with an X-ray tube voltage of 50 kV, current of 200 μA, rotation step of 0.40°, at a scanning voxel size of 5.5 μm and with the use of an aluminium filter (0.5 mm in thickness). The scanning total angular rotation was 185° with an angular increment of 0.4°. Datasets were reconstructed using cone-beam reconstruction software (Skyscan NRecom) based on the Feldkamp algorithm [24] and segmented into binary images using adaptive local thresholding. Every pixel of the reconstructed 8-bit BMP images has a colour or grey value between 0 (black) and 255 (white). Morphometric indices were determined from the microtomographic datasets (integrated over a volume of interest, VOI) using direct 3D morphometry. Bone regions were obtained by free drawing regions of interest and analyzed using the commercial software provided with the equipment (SkyScan™ CT-analyzer software, version 1.7.0). For trabecular bone analysis at the metaphysis, a VOI was selected starting at a distance of 1.00 mm from the growth plate of tibial proximal metaphysis and extending a further longitudinal distance of 4.00 mm in the distal direction excluding cortical bone. For subchondral bone analysis, a VOI was selected between the subchondral plate and the growth plate of the proximal metaphysis. Thresholding was applied to the images to segment the trabecular bone from the background and the same threshold setting was used for all the samples. Morphometric indices were determined from the microtomographic data sets (integrated over a VOI) using direct 3D morphometry. Total volume of VOI (tissue volume; TV; mm^3^) and trabecular bone volume (BV; mm^3^) were calculated based on the hexahedral marching cubes volume model of the VOI. Trabecular bone volume (BV/TV; %) was directly calculated. Trabecular thickness (Tb.Th; mm), trabecular separation (TbS; mm) and trabecular number (Tb.N; 1/mm) were measured directly on 3D images. Measurements of Tb.Th were calibrated by scanning and analyzing three aluminum foils with thicknesses of 50, 125 and 250 μm. The non-metric indices, such as trabecular bone pattern factor (Tb.Pf; 1/mm) were also calculated using the direct 3D model.

### Determination of inflammatory mediators

The knees were amputated, skin was dissected and the rest of tissue was immediately frozen in liquid nitrogen and pulverized in a Freezer/Mill 6750 (Spex SamplePrep, Metuchen, NJ, USA). The powder was extracted with 2 ml of A buffer pH 7.4 [10 mM 4-(hydroxyethyl)-1-piperazine-ethane-sulfonic acid (HEPES, pH 8), 1 mM EDTA, 1 mM ethylene glycol bis(β-aminoethylether)-N,N,N’,N’-tetraacetic acid (EGTA), 10 mM KCl, 1 mM dithiothreitol, 5 mM NaF, 1 mM Na_3_VO_4_, 1 μg/ml leupeptin, 0.1 μg/ml aprotinin and 0.5 mM phenylmethylsulfonyl fluoride]. After the first centrifugation at 1,200 g (10 min at 4°C), supernatants were collected, centrifuged at 9,000 g (15 min at 4°C) and used to measure cytokines by ELISA. Interleukin(IL)-1β and tumour necrosis factor-α (TNFα) were measured by using the kits from R&D Systems (Minneapolis, MN, USA) with a sensitivity of 5.0 pg/ml, and IL-6 and IL-17A with the kits from eBioscience (San Diego, CA, USA) with a sensitivity of 12.0 and 1.0 pg/ml, respectively. Prostaglandin E_2_ (PGE_2_) was determined by radioimmunoassay [25].

### Determination of serum biomarkers

Serum was used for the determination by ELISA of C-telopeptide of type II collagen (CTX-II, serum pre-clinical Cartilaps, Nordic Biosciences, Herlev, Denmark, sensitivity of 6.3 pg/ml), cartilage oligomeric matrix protein (COMP, MD Biosciences, Zürich, Switzerland, sensitivity <0.2 U/l), matrix metalloproteinase (MMP)-3 (R&D Systems, sensitivity of 19.0 pg/ml), N-terminal propeptide of type I procollagen (PINP, IDS Inc., Boldon, UK, sensitivity of 0.7 ng/ml) and tartrate-resistant acid phosphatase (TRAP)-5b (sensitivity of 0.1 U/l). Serum levels of osteocalcin (OC), osteoprotegerin (OPG) and receptor activator of nuclear factor κB ligand (RANKL) were determined by luminex, with sensitivity of 7.0, 2.3 and 3.3 pg/ml, respectively (Millipore Corporation, Billerica, MA, USA). Alkaline phosphatase (ALP) levels in serum were determined as previously described [26].

### Statistical analysis

The results are presented as mean ± standard deviation (SD), n: number of animals. The level of statistical significance was determined by using one-way analysis of variance (ANOVA) followed by Bonferroni’s test. Scoring was analyzed by a non-parametric test (Kruskal-Wallis followed by Dunn's post-test).

## Results

### Effect of BIS076 on histopathological changes in the joint

**Figure 1:**
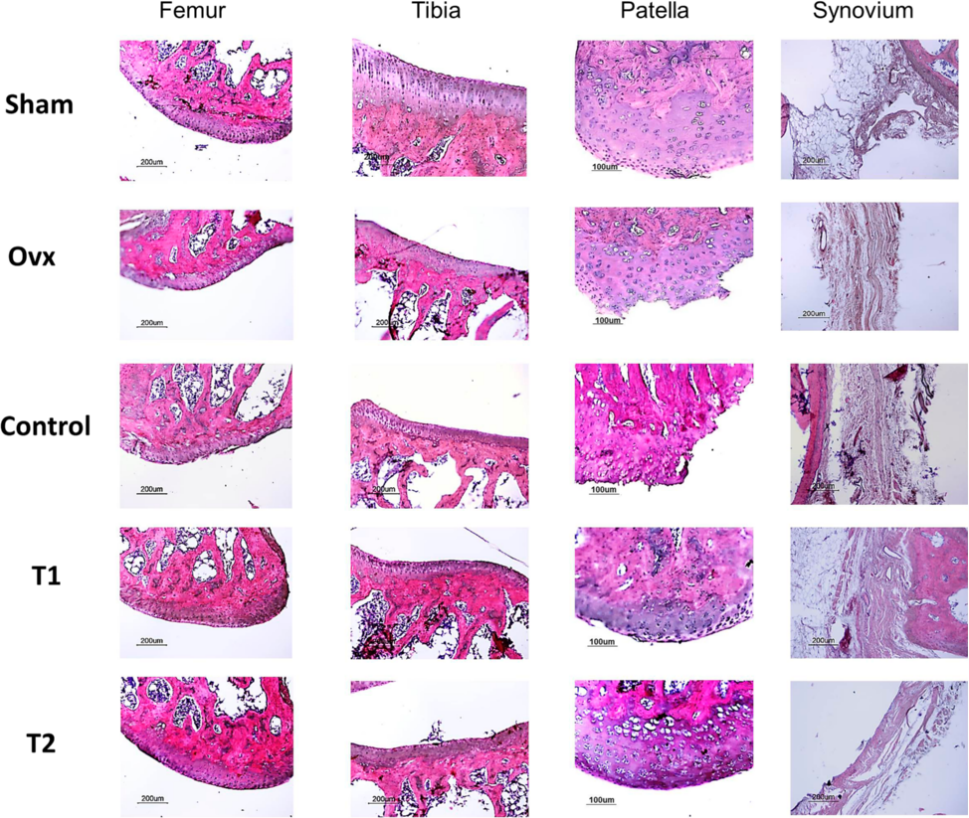
Histochemical analysis in the ACLT knees. Representative images of sections from femur, tibia, patella stained with haematoxylin and eosin. Bars: 200 μm and 100 μm.

**Figure 2:**
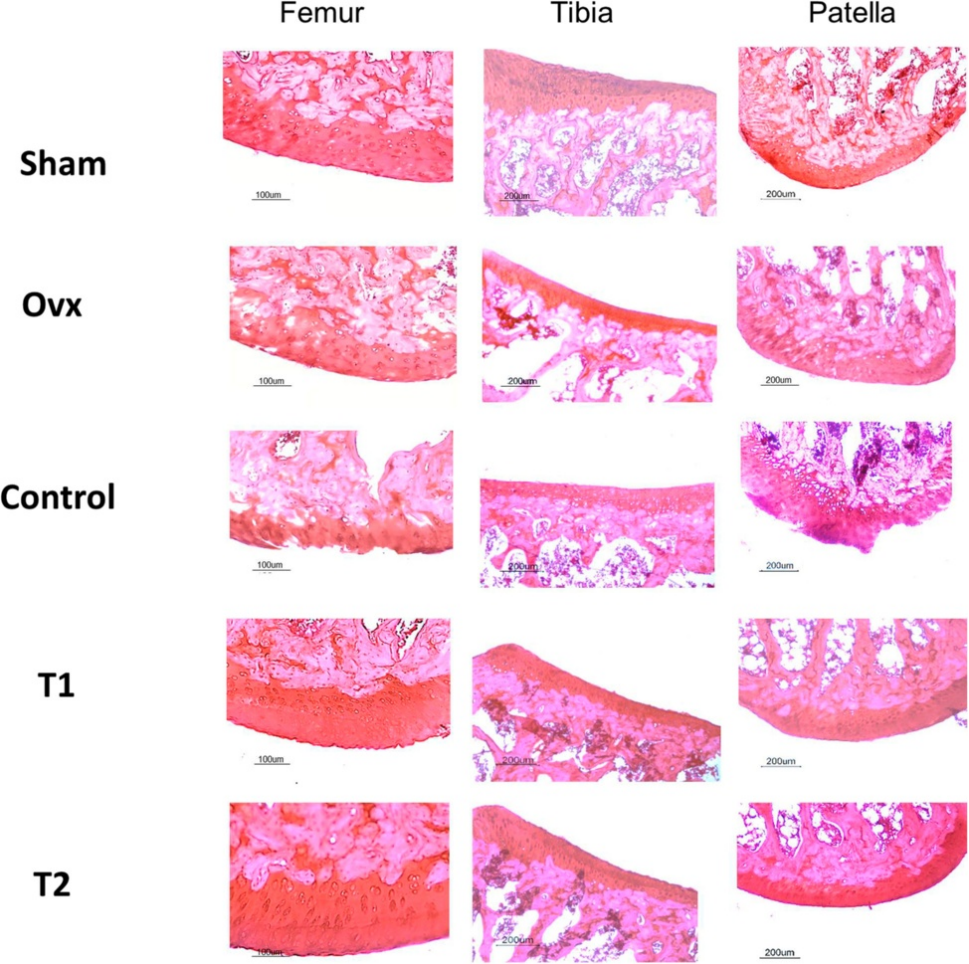
Histochemical analysis in the ACLT knees. Representative images of sections from femur, tibia, patella stained with safranin O. Bars: 200 μm and 100 μm.

**Figure 3:**
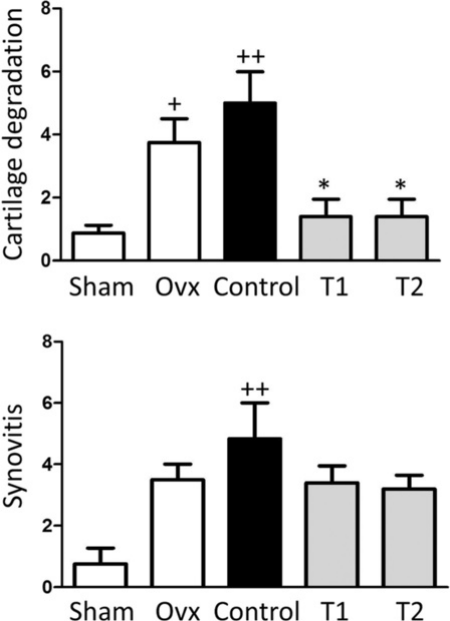
Cartilage degradation and synovitis were scored in frontal sections of knee joints as indicated in materials and methods. Cartilage degradation was determined in accordance with the Osteoarthritis Research Society International (OARSI) histopathology grading and staging system [22]. Synovitis was scored according to Krenn *et al*. [23]. Results are expressed as mean ± SD (n = 5); ^++^ *P* < 0.01 with respect to Sham; *P < 0.05 compared to Control (Kruskal-Wallis followed by Dunn's post-test).

The histopathological analysis showed the presence of cartilage damage in Control rats (Figures 1 and 2). Structural changes in cartilage surface included focal damage and fibrillation, loss of proteoglycan, decreased chondrocyte cell number and increased chondrocyte clustering. These pathological changes were reflected by the score of the joint in Control group which was significantly higher than the sham group (Figure 3). The grade of cartilage damage in the groups treated with BIS076 (T1 and T2) was significantly lower than in Control group and not statistically different from the sham-operated group. In addition, Control rats showed synovitis which was scored according to Krenn *et al*. [23]. In rats treated with BIS076 at both doses (T1 and T2), a lower level of synovitis was observed compared with Control animals although it did not reach statistical significance.

### Effect of BIS076 on bone micro-architecture

**Figure 4:**
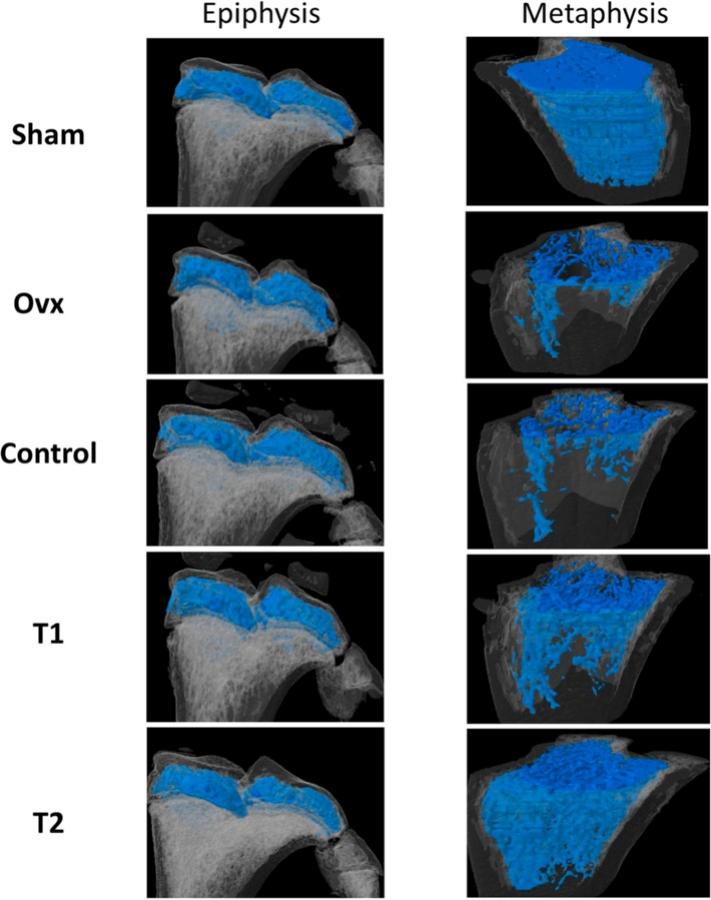
Representative images of epiphysis and metaphysis of tibia by μCT analysis. Quantification of trabecular bone parameters in both regions is shown in Table 2.

μCT analysis indicated loss of bone mass and structural alterations in Ovx and Control rats compared with Sham animals (Table 2 and Figure 4). Main bone changes were observed in metaphysis, with significant reductions in bone volume fraction (BV/TV), bone surface density (BS/TV), trabecular number (Tb.N) and volumetric bone mineral density (vBMD) whereas trabecular factor (Tb.Pf) significantly increased. Similar changes were observed in the parameters measured in epiphysis (Ovx or Control versus Sham) although they did not reach statistical significance. Interestingly, BIS076 counteracted all bone alterations shown in Control animals in the metaphysis only, with statistically significant effects at the higher dose (T2).

**Table 2:** **Effect of BIS076 on bone parameters after μCT analysis**

**Metaphysis**					
	**Sham**	**Ovx**	**Control**	**T1**	**T2**
BV/TV(%)	42.0 ± 11.5	12.6 ± 4.8^++^	9.7 ± 2.1^++^	15.1 ± 1.0	29.6 ± 7.7**
BS/TV(mm^−1^)	14.2 ± 0.4	4.9 ± 1.4^++^	4.8 ± 0.8^++^	6.7 ± 0.4	12.6 ± 0.6*
Tb.Th(μm)	102.7 ± 9.6	98.5 ± 6.6	94.3 ± 1.7	93.9 ± 1.6	96.6 ± 2.0
Tb.N(mm^−1^)	4.0 ± 0.3	1.2 ± 0.4^++^	1.2 ± 0.2^++^	1.7 ± 0.1	3.3 ± 0.1*
Tb.Pf(mm^−1^)	0.03 ± 0.02	12.1 ± 3.3^++^	15.0 ± 0.8^++^	10.6 ± 2.0	4.7 ± 2.2*
vBMD(mg/cm^3^)	413.0 ± 65.1	N.D.	89.8 ± 30.3^++^	112.6 ± 23.5	154.1 ± 40.1*
**Epiphysis**					
	**Sham**	**Ovx**	**Control**	**T1**	**T2**
BV/TV(%)	40.3 ± 14.7	34.3 ± 1.7	32.3 ± 0.3	34.3 ± 1.6	33.8 ± 0.1
BS/TV(mm^−1^)	12.2 ± 0.9	10.7 ± 0.2	10.6 ± 0.6	10.7 ± 1.1	11.7 ± 0.4
Tb.Th(μm)	127.8 ± 1.7	115.2 ± 4.0	115.6 ± 2.9	120.3 ± 1.8	117.9 ± 3.9
Tb.N(mm^−1^)	3.4 ± 0.5	2.9 ± 0.1	2.9 ± 0.2	2.8 ± 0.3	3.1 ± 0.1
Tb.Pf(mm^−1^)	0.02 ± 0.02	5.0 ± 1.0^++^	5.6 ± 0.6	3.8 ± 0.4	4.2 ± 1.2
vBMD(mg/cm^3^)	499.9 ± 107.4	434.6 ±15.3	431.0 ± 24.4	430.7 ± 66.1	467.6 ± 54.7

Results are shown as mean ± SD (n = 3). ^++^ *P* < 0.01 with respect to Sham; *P < 0.05, **P < 0.01 with respect to Control (ANOVA followed by Bonferroni’s test). N.D. not determined.

Three-dimensional trabecular microarchitecture was analyzed at the metaphysis and epiphysis of the tibia.

### Effect of BIS076 on inflammatory mediators in the joint

**Figure 5:**
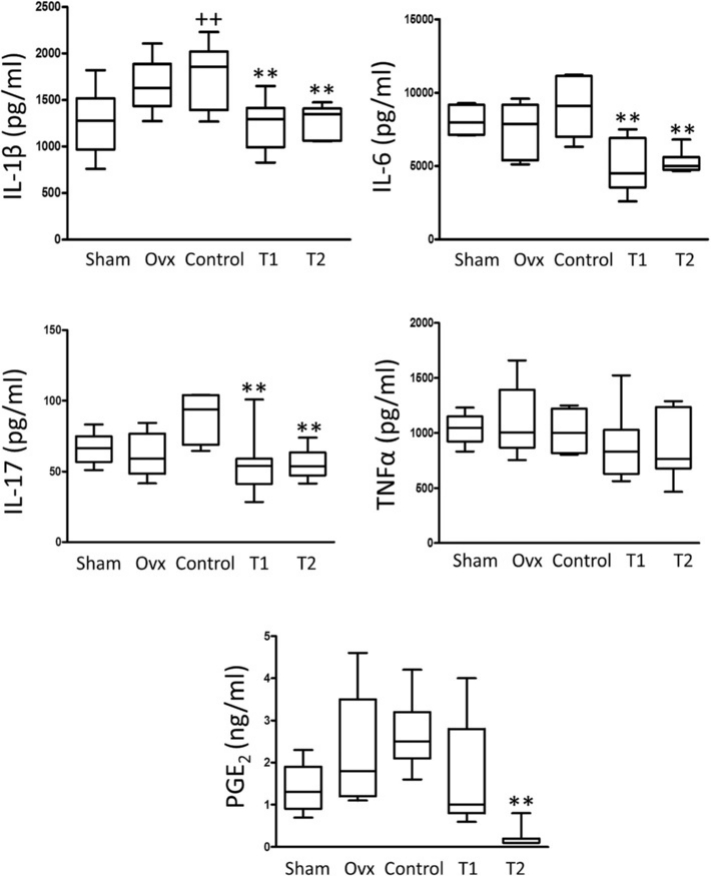
Levels of pro-inflammatory mediators in homogenates from ACLT knees. IL-1β, IL-6, IL-17 and TNFα were measured by ELISA. PGE_2_ was measured by radioimmunoassay. Results show mean ± SD (n = 7); ^+^ *P* < 0.05 with respect to Sham; *P < 0.05, **P < 0.01 compared to Control (ANOVA followed by Bonferroni’s test).

We have shown previously the contribution of inflammation to the progression of OA after ACLT [27]. The levels of the inflammatory mediators IL-1β, IL-6, IL-17, TNFα and PGE_2_ were determined in knee homogenates. Figure 5 shows that IL-1β, IL-17 and PGE_2_ were significantly higher in the Control compared with the Sham group. Treatment with BIS076 reduced the expression of inflammatory mediators with significant effects for T1 and T2 on cytokines IL-1β, IL-6 and IL-17, and for T2 on PGE_2_.

### Effect of BIS076 on serum biomarkers

**Figure 6:**
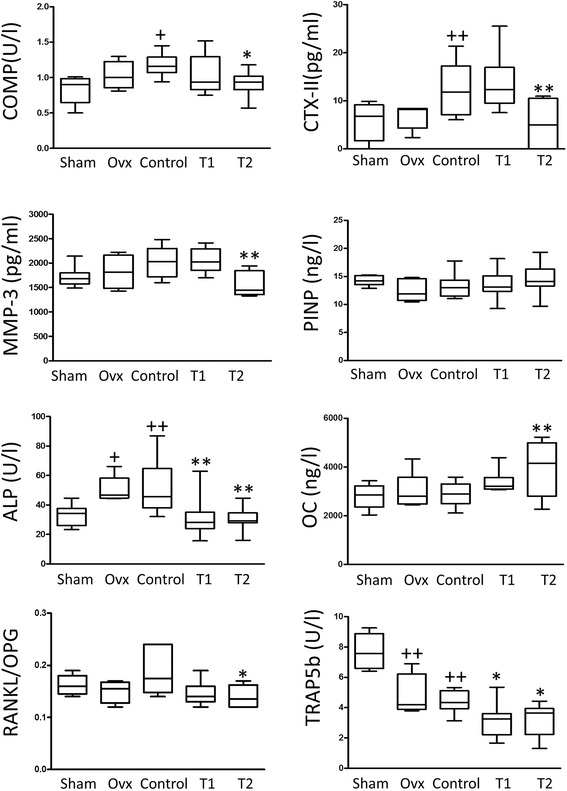
Levels of serum biomarkers. COMP, CTX-II, MMP-3, PINP and TRAP-5b were measured by ELISA. Serum levels of OC, OPG and RANKL were determined by luminex. ALP levels in serum were determined as previously described [26]. Results show mean ± SD (n = 12-15); ^+^ *P* < 0.05, ^++^ *P* < 0.01 with respect to Sham; *P < 0.05, **P < 0.01compared to Control (ANOVA followed by Bonferroni’s test).

The cartilage degradation markers CTX-II and COMP were measured in serum. Control animals showed enhanced levels of these molecules with respect to sham-operated rats (Figure 6). BIS076 reduced the expression of cartilage degradation markers with a significant effect at the higher dose (T2). MMP-3, an inflammatory marker related with cartilage destruction in OA [28] was also assessed. The levels of MMP-3 observed in Control rats were significantly reduced by BIS076 treatment at the higher dose (T2). The levels of bone turnover markers were also determined. The levels of ALP significantly increased and those of TRAP5b decreased in the groups Ovx and Control. BIS076 at both doses (T1 and T2) significantly reduced the increase of ALP activity shown in Control rats, as well as TRAP5b levels and the RANKL/OPG ratio whereas OC was significantly enhanced by the higher dose (T2) of BIS076.

## Discussion

Disruption of the balance between degradative and repair processes leads to loss of cartilage integrity in OA. Cartilage degeneration follows joint instability after ACLT and it is associated with localized destruction of the type II collagen network [29]. In this study, OA was induced by ACLT in ovariectomised rats. This experimental model allows the evaluation of treatments able to act on joint degradation and bone alterations related to OA and osteoporosis. We analyzed the effects of BIS076, a new formulation of a natural porcine cartilage extract associated with hydroxyapatite and vitamin D_3_ and found that important alterations in cartilage, synovial membrane and bone induced in Control rats were improved in animals treated with BIS076.

Radiographic severity of knee OA is associated with serum or urinary levels of cartilage turnover markers such as CTX-II and COMP [30,31]. In this experimental model, these biomarkers were increased in the serum of Control rats with respect to sham-operated animals at the end of the experiment. The protection exerted by BIS076 against cartilage degradation was reflected by the reduction in the levels of CTX-II and COMP. In addition, BIS076 down-regulated MMP-3, an inflammatory marker that may be a predictor of radiographic OA progression [28].

Cartilage homeostasis is maintained at the normal joint by a balance between anabolic and catabolic processes. Nevertheless, catabolic events leading to cartilage destruction predominate in OA. Overloading and inflammation are factors contributing to knee OA. It is accepted that inflammatory changes mediate the progression of joint lesion [32,33] and activation of chondrocytes by mechanical stress results in the production of inflammatory mediators. In fact, synovitis and inflammatory mediators such as cytokines and chemokines are present in early-stage and last-stage disease. Pro-inflammatory cytokines induce catabolic enzymes and cartilage matrix degradation [34] leading to an imbalance between cartilage matrix degradation and repair. In turn, cartilage matrix molecules and degradation products can induce inflammatory responses creating a vicious circle [35]. In addition, pro-inflammatory cytokines induce cyclooxygenase-2 and microsomal PGE synthase-1 in articular tissues leading to an enhanced production of PGE_2_ that may contribute to synovitis and cartilage degradation [36,37]. Our data indicate that BIS076 lowers the production of pro-inflammatory cytokines and PGE_2_ in the affected joint. Although further studies would be necessary to determine the mechanism of action of this formulation, it is likely that its anti-inflammatory effects contribute to the reduction in OA progression.

On the other hand, trabecular bone alterations in metaphysis were improved by BIS076, with increases in volumetric bone mineral density, bone volume fraction, bone surface density, trabecular number and connectivity. These effects were related to the modification of bone metabolism by BIS076 which partly counteracted the increase in ALP activity shown in Control rats and enhanced OC levels at the highest dose. Interestingly, this formulation reduced the RANKL/OPG ratio, an index of osteoclastic activation [38,39], and the levels of the resorption marker TRAP5b, suggesting that the protective effects of BIS076 are related to a decreased trabecular bone resorption.

The different components of BIS076 may contribute to the observed effects. In addition to the well-known role of vitamin D_3_ and calcium in bone metabolism, some studies have suggested a protective effect of this vitamin on OA [40]. Nevertheless, clinical trials of vitamin D supplementation have failed to show any reduction in knee pain or cartilage volume loss in patients with symptomatic knee OA [41]. Therefore, it is likely that collagen and glycosaminoglycan components of BIS076 play a main role in experimental OA. It is interesting to note that hydrolysed collagen enhances the synthesis of type II collagen by bovine chondrocytes [42] and clinical studies have suggested that oral ingestion of this product reduces pain in OA patients and it may have an additive effect with calcitonin on osteoporosis [43]. Furthermore, previous reports have suggested that chondroitin sulfate exerts some anabolic effects on cartilage and also inhibits nuclear factor-κB activation leading to the down-regulation of inflammatory mediators and catabolic enzymes such as MMPs thus preventing cartilage degradation [44].

## Conclusions

This study has demonstrated that BIS076 significantly reduces the severity of structural changes in joint tissues, down-regulates the inflammatory response and improves metaphyseal bone density and micro-architecture in a model of OA induced by ACLT in ovariectomised rats. These findings suggest that BIS076 is a new promising and effective formulation for OA and could have a potential role in bone protection during treatment.

## Abbreviations

ACLT: Anterior cruciate ligament transection
ALP: Alkaline phosphatase
ANOVA: Analysis of variance
COMP: Cartilage oligomeric matrix protein
CTX-II: C-Telopeptide of type II collagen
IL: Interleukin
MMP: Matrix metalloproteinase
OA: Osteoarthritis
OARSI: Osteoarthritis research society international
OC: Osteocalcin
OPG: Osteoprotegerin
PINP: N-terminal propeptide of type I procollagen
PGE_2_: Prostaglandin E_2_
RANKL: Receptor activator of nuclear factor κB ligand
TNFα: Tumor necrosis factor-α
TRAP: Tartrate-resistant acid phosphatase.
